# The Role of Macroautophagy and Chaperone-Mediated Autophagy in the Pathogenesis and Management of Hepatocellular Carcinoma

**DOI:** 10.3390/cancers14030760

**Published:** 2022-02-01

**Authors:** Anastasia D. Karampa, Anna C. Goussia, Georgios K. Glantzounis, Eleftheria M. Mastoridou, Nikolaos-Andreas T. Anastasopoulos, Antonia V. Charchanti

**Affiliations:** 1 HPB Unit, Department of Surgery, Faculty of Medicine, School of Health Sciences, University of Ioannina, 45500 Ioannina, Greece; nata_kar2007@yahoo.gr (A.D.K.); gglantzounis@uoi.gr (G.K.G.); n.anastasopoulos@uoi.gr (N.-A.T.A.); 2 Department of Pathology, Faculty of Medicine, School of Health Sciences, University of Ioannina, 45500 Ioannina, Greece; agoussia@uoi.gr; 3 Department of Anatomy-Histology-Embryology, Faculty of Medicine, School of Health Sciences, University of Ioannina, 45500 Ioannina, Greece; eleftheriamast95@gmail.com

**Keywords:** hepatocellular carcinoma, macroautophagy, chaperone-mediated autophagy, chemoresistance

## Abstract

**Simple Summary:**

Hepatocellular carcinoma (HCC) is a major health problem with the second highest mortality among all cancers and a continuous increase worldwide. HCC is highly resistant to available chemotherapeutic agents, leaving patients with no effective therapeutic option and a poor prognosis. Although an increasing number of studies have elucidated the potential role of autophagy underlying HCC, the complete regulation is far from understood. The different forms of autophagy constitute important cell survival mechanisms that could prevent hepatocarcinogenesis by limiting hepatocyte death and the associated hepatitis and fibrosis at early stages of chronic liver diseases. On the other hand, at late stages of hepatocarcinogenesis, they could support the malignant transformation of (pre)neoplastic cells by facilitating their survival.

**Abstract:**

Hepatocarcinogenesis is a long process with a complex pathophysiology. The current therapeutic options for HCC management, during the advanced stage, provide short-term survival ranging from 10–14 months. Autophagy acts as a double-edged sword during this process. Recently, two main autophagic pathways have emerged to play critical roles during hepatic oncogenesis, macroautophagy and chaperone-mediated autophagy. Mounting evidence suggests that upregulation of macroautophagy plays a crucial role during the early stages of carcinogenesis as a tumor suppressor mechanism; however, it has been also implicated in later stages promoting survival of cancer cells. Nonetheless, chaperone-mediated autophagy has been elucidated as a tumor-promoting mechanism contributing to cancer cell survival. Moreover, the autophagy pathway seems to have a complex role during the metastatic stage, while induction of autophagy has been implicated as a potential mechanism of chemoresistance of HCC cells. The present review provides an update on the role of autophagy pathways in the development of HCC and data on how the modulation of the autophagic pathway could contribute to the most effective management of HCC.

## 1. Overview of Autophagy

Autophagy is an evolutionarily conserved self-digestion process whereby misfolded macromolecules and dysfunctional organelles (referred to as “autophagic cargo”) undergo lysosomal degradation [1]. Investigations of autophagy have demonstrated that various signals could trigger its activation. Autophagy exists under basal conditions (“basal autophagy”), with a significant role in the elimination of damaged proteins and organelles to maintain cellular quality control [2,3]. However, autophagy is also considered an adaptive process as it could be induced under stressful conditions (“stress-induced autophagy”) by different-molecular signaling pathways in response to stimuli, such as energy deprivation, hypoxia, and inflammation, thereby sustaining cell metabolism and survival [2,3,4,5]. There are three main types of autophagy: macroautophagy, microautophagy, and chaperone-mediated autophagy (CMA), all of which differ in the lysosomal transfer of the autophagic cargo [3]. Considering macroautophagy, misfolded proteins and dysfunctional organelles are sequestered in autophagosomes, which are then delivered for lysosomal degradation [6]. During microautophagy, the lysosomes directly engulf small autophagic substrates by late endosomes forming multivesicular bodies and fusing with the lysosome for degradation [7]. In contrast, CMA relies on selective recognition of cytosolic proteins characterized by a pentapeptide amino acid motif (KFERQ), which are then internalized into the lysosomes for degradation through direct interaction with the heat shock cognate protein 70 (Hsc70) [8].

Numerous studies have reported the role of autophagy in the liver both under physiological and pathological conditions, emerging as a crucial regulator of various liver diseases [9]. The liver is a complex organ with a heterogeneous cellular architecture characterized by high metabolic needs, which render it primarily dependent on the autophagy mechanism to maintain homeostasis and ensure proper function [10]. Under normal conditions, basal levels of autophagy serve as a mechanism to eliminate dysfunctional organelles and protein aggregates through lysosomal degradation, thus maintaining energy homeostasis [11,12]. However, disturbance of basal autophagy can lead to accumulation of dysfunctional molecules, such as damaged mitochondria and distorted peroxisomes leading to liver injury [13,14]. On the other hand, stress-induced autophagy serves as a substrate-supplying and energy-providing mechanism via recycling of cellular molecules, thus providing energy and contributing to cell survival under hostile conditions, such as tumor microenvironment [15].

## 2. Autophagy Pathways Involved in Liver Pathophysiology

Under basal conditions, macroautophagy is an integrated mechanism of hepatic homeostasis and energy balance through the degradation of aggregated proteins, therefore preserving cellular quality control and the recycling of proteins, thus contributing to the constant enrichment of the amino-acid pool and the adequate coverage of metabolic needs [16].

**Figure 1:**
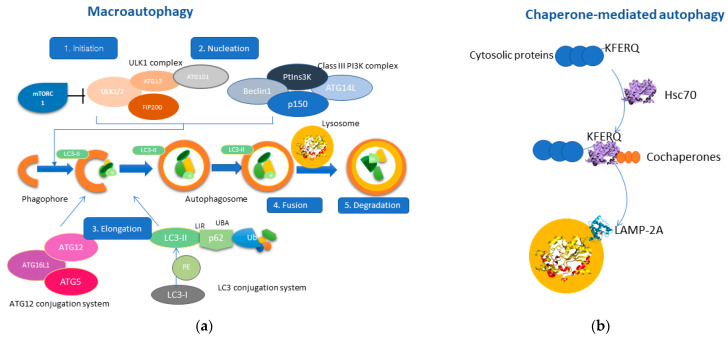
Molecular pathways of macroautophagy and chaperone-mediated autophagy (CMA). (**a**) Macroautophagy consists of five main stages; initiation consists of activating the ULK1 complex after inhibition of mTORC1. Nucleation includes the interaction of the ULK1 complex with the Class III PI3K complex, promoting the formation of the phagophore. Elongation includes the interaction between the ATG-12 conjugation system with the LC3-conjugation system by promoting the bending of the phagophore membrane and resulting in the formation of the autophagosome, which engulfs the autophagic cargo. Stage four consists of the fusion of the autophagosome with the lysosome leading to the degradation of the sequestered autophagic cargo through lysosomal hydrolases. (**b**) CMA is a particular type of autophagy, which involves the interaction of HSC70 with cytosolic proteins containing the KFERQ motif. This complex also contains other co-chaperones, thereby mediating the translocation to the lysosomal membrane. LAMP-2A is a receptor protein residing in the lysosomal membranes which interact with the HSC70-protein complex. The multimerization of LAMP-2A results in the sequestration of the autophagic cargo into the lysosomal lumen and the subsequent degradation.

Macroautophagy consists of five closely related steps: initiation, nucleation, elongation, fusion, and degradation [17]. Mammalian target of rapamycin (mTOR) is the primary regulator of autophagy [18], as inhibition of the mTOR signaling pathway by various signals (i.e., energy deprivation, stressful stimuli) results in activation of autophagy (stage 1: Initiation) [19]. The second stage (stage 2: Nucleation) includes two protein complexes, ULK1 complex [20] and the Class III PI3K complex (Beclin1, ATG14, VPS34, p150), which interact in the formation of the phagophore. Consequently, the phagophore membrane closes through the interaction of the ATG12 conjugation system and the LC3 conjugation system (stage3: Elongation) [21,22]. Thus, the phagophore finally forms the autophagosome, which will ultimately engulf cytosolic material as autophagic cargo [1]. The interaction between the growing autophagosome membrane and the autophagic cargo is mediated through the LC3-II protein, which acts as a “receptor” on the phagophore, and interacts with p62/SQSTM1, a multifunctional adaptor protein that recognizes ubiquitin molecules on the autophagic cargo [23]. Once the autophagosome is formed, it fuses with the lysosome membrane (stage 4: Fusion) [24] resulting in the release of the autophagic cargo into the lysosomal lumen, where it will be degraded by lysosomal hydrolases (stage 5: Degradation) [25]. The molecular mechanism of macroautophagy is represented in Figure 1.

Macroautophagy seems to constitute the major contributor to autophagic flux in the liver under normal conditions. However, studies have shown that HCC tissue is characterized by the accumulation of p62, thus indicating that macroautophagy is inhibited in HCC, since p62 expression levels are inversely correlated with autophagic activity [26]. On the contrary, Chava et al. have provided evidence that CMA is upregulated as a counterbalanced mechanism for the impairment of macroautophagy to promote HCC survival [27]. Thus, this study highlights the compensatory role of CMA in HCC development when macroautophagy is impaired, results that are similar to previous reports showing that increased CMA activity is needed for tumor cell survival [28,29].

The CMA process, which is characterized by cargo selectivity, is coordinated through five main steps. The first step involves the selection of specific soluble cytosolic proteins, which contain a consensus pentapeptide known as the KFERQ-like motif [30,31]. The second step is the recognition of these proteins by Hsc70 cytosolic chaperone, which interact with other co-chaperones (i.e., heat shock protein 40 (HSP-40), HSP70, HSP90), that contribute to the translocation of the autophagic substrate into the lysosomal membrane [32]. CMA is distinct from the other types of autophagy as it does not require any type of membranous vesicles. In fact, the third step includes binding of the protein–chaperone complex to the lysosome membrane through direct interaction with the lysosome-associated membrane protein type 2A (LAMP-2A), which serves as a receptor of autophagic components [30]. Docking of the protein substrate to LAMP-2A triggers its multimerization, which is necessary for the subsequent translocation of the autophagic substrate into the lysosomal lumen. Hsc70 does not enter the lysosomal lumen for degradation, as it remains in the cytosolic compartment to exert its physiological role. In fact, substrate internalization is mediated by a lysosomal form of Hsc70 (lys-Hsc70) and is followed by degradation of the autophagic cargo and subsequent disassembly of LAMP-2A from the complex (fifth step) [33]. Interestingly, the levels of LAMP-2A expression directly correlate with CMA activity, which could be enhanced or downregulated depending on the surrounding cellular conditions [34]. The molecular mechanism of the CMA pathway is depicted in Figure 1.

## 3. Role of Macroautophagy and CMA in HCC Development

**Figure 2:**
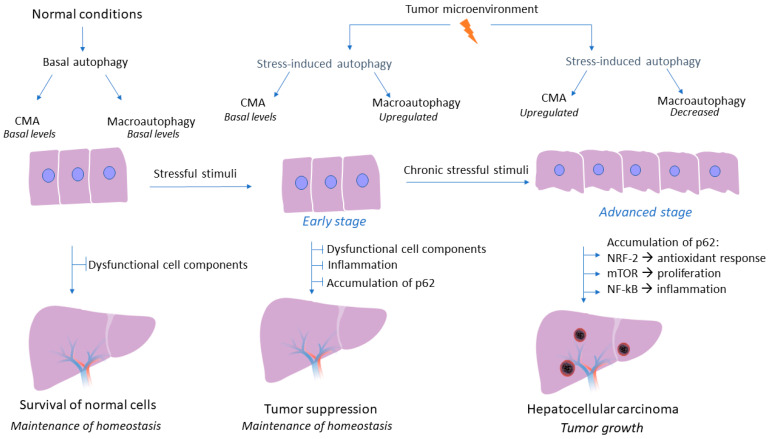
Role of autophagy mechanism during hepatocarcinogenesis. Under non-pathogenic conditions, both macroautophagy and CMA are activated at basal levels, while the central role of their autophagy flux is the degradation of dysfunctional cell components and the maintenance of liver homeostasis. During the early stages of hepatocarcinogenesis macroautophagy is the first to sense energy deprivation. The adverse conditions that characterize tumor microenvironment induce macroautophagy activity which acts as a tumor suppressor mechanism by promoting the degradation of dysfunctional cell components and by preventing inflammation and accumulation of p62 under stressful conditions. Nonetheless, during later stages of HCC development CMA seems to be upregulated under continuous severe stressful stimuli and when the tumor is established as a potential compensatory mechanism to impaired macroautophagy. However, during the advanced stage of HCC development, CMA pathway is upregulated as a response to continuous stressful conditions and prolonged periods of nutrient deprivation that characterize tumor microenvironment. On the contrary, macroautophagy decreases subsequently, as established HCC liver tissue is characterized by accumulation of p62, a biomarker of macroautophagy activity. Therefore, CMA seems to act as a potential compensatory mechanism to impaired macroautophagy. Downregulation of macroautophagy and subsequent accumulation of p62, results in activation of NRF-2, thus promoting a constant antioxidant response. Moreover, p62 activates the mTOR signaling pathway, which results in tumor cell proliferation and growth regardless of growth factors levels, while p62 also promotes NF-kB activation, thus promoting inflammatory conditions, a cardinal feature of tumor growth.

Although the crucial role of autophagy flux in liver homeostasis and regulation of hepatic response during pathological conditions has been well established, the tumor-promoting or tumor-suppressive role of macroautophagy and CMA pathway in HCC is still a controversial research topic. A wide variety of studies have elucidated the effects of autophagy on HCC, with their conflicting results being more than evident. Macroautophagy could act as a tumor suppressor factor, especially in the early stages of tumorigenesis, by limiting inflammation and inhibiting the accumulation of p62 and dysfunctional cellular components, thus preventing tumor initiation [35]. A study on animal models deficient in macroautophagy has demonstrated the subsequent accumulation of dysfunctional organelles and damaged proteins that could potentially lead to elevated levels of reactive oxygen species, inflammatory conditions, and genomic instability [36]. On the contrary, considerable research data has shown that in established tumors, the autophagy mechanism could act as a tumor promoter by enhancing the survival of tumor cells. In fact, the hostile tumor microenvironment, characterized by energy deficit and oxygen deprivation, triggers autophagy mechanisms to provide nutrient supplements and degrade toxic substances that could potentially lead to cell death of tumor cells [37]. However, these studies had not examined whether the autophagy pathway concerns macroautophagy or CMA, as there is emerging evidence that macroautophagy is dysregulated during HCC development and CMA acts as a compensator mechanism for the impairment of macroautophagy to promote HCC development [27]. In fact, CMA is responsible for the degradation of 30% of cytosolic proteins under stress conditions; therefore, future investigations could help us gain further insight into whether CMA is also responsible for the degradation of autophagic molecules that contribute to macroautophagy inhibition [38]. Intriguingly, a recent study has demonstrated that CMA activation promotes a LAMP-2A-associated Βeclin 1 degradation in the lysosomes, in a persistently infected hepatitis C virus cell culture, thereby indicating that CMA could inhibit macroautophagy via degradation of specific autophagic molecules [39]. Taken together, the autophagy mechanism may act both as a promoter and as an inhibitor of tumorigenesis, a role which highly depends on the molecular pathway (macroautophagy or CMA) and the stage of carcinogenesis (early or advanced stage), thereby, investigating in depth the interaction of autophagy mechanism with the tumor microenvironment and the mechanisms underlying the controversial effects of autophagy, is essential in order to utilize autophagy-related molecules as an effective and promising therapeutic approach in HCC. The complex role of autophagy in HCC is presented in Figure 2.

### 3.1. Upregulation of Macroautophagy Acts as a Tumor-Suppressing Mechanism in Early Stages of HCC

Macroautophagy has been associated with tumor-suppressive properties, especially in the early stages of carcinogenesis. This type of autophagy serves as a quality control cellular checker, promotes genomic stability, prevents chronic inflammation and thus chronic tissue injury, and inhibits the accumulation of oncogenic p62 protein [40,41]. Sun et al. have demonstrated that macroautophagy inhibition in animal models promotes hepatocarcinogenesis in the dysplastic stage, while suppresses tumorigenesis in the tumor-forming stage [42]. Furthermore, dysregulation of macroautophagy has been shown to affect the progression of numerous liver diseases, which are considered high-risk factors for HCC [40,41]. The macroautophagy pathway is deeply involved in numerous precancerous etiologic factors and in the development of HCC itself, suggesting that it is considered a crucial and multifaceted mechanism of liver pathogenesis [41].

Various studies have elucidated the protective role of macroautophagy against liver tumorigenesis. Ding et al. initially reported that macroautophagy defects in the human liver contribute to HCC development [43]. In fact, in vivo studies in animal models have demonstrated that genetic depletion or dysfunction of basic macroautophagy related proteins, such as Beclin 1, has increased frequency for premalignant lesions and cancer [44,45,46]. In addition, Beclin 1 has been found downregulated in human HCC [43,47], while Beclin 1 deletion has been associated with the development of HBV-related HCC [44]. Interestingly, levels of Beclin-1 have been investigated as an independently predicted marker of HCC tumor progression, significantly associated with disease-free survival and overall survival rates [43,48].

Furthermore, in vivo studies in mice models with a deletion in Atg5 and Atg7, two key macroautophagy genes, have shown increased incidence for malignancies, notably only in the liver, suggesting the high sensitivity and dependence of the liver homeostasis on autophagy pathways [13,49]. Somatic mutations of ATG5 protein have been found in several human tissue samples of HCC [50]. The tumor-suppressive role of macroautophagy is probably associated with its ability to eliminate protein aggregates, mutated proteins, and dysfunctional organelles [51]. The failure to degrade all the above-damaged molecules promotes chronic inflammation, oxidative stress, and genomic instability, leading to malignant transformation and the development of HCC [52].

Chronic inflammation is a well-documented carcinogenic factor [53]. However, macroautophagy can keep excessive inflammation at bay and control inflammatory signaling pathways. Further investigations in the molecular interaction between macroautophagy and inflammation in HCC have shown that macroautophagy can regulate inflammation via damage-associated molecular patterns (DAMPs) [54]. When the cell is exposed to DAMPs, mitophagy, a selective type of macroautophagy, prevents the accumulation of mitochondrial reactive oxygen species (ROS) and DNA from avoiding over-activation of inflammation [55]. Inflammatory cytokines can also affect macroautophagy levels, thus suggesting a bidirectional regulation between the two pathways. Studies have demonstrated that the effect of cytokines on autophagy is complex and may depend on cell types [56].

The anti-inflammatory role of macroautophagy is further elucidated by studies indicating that dysfunctional macroautophagy leads to the accumulation of damaged proteins and organelles, stimulating inflammatory conditions [54]. Under these conditions, a microenvironment characterized by elevated oxidative stress hypoxia and various stressful stimuli promotes genomic instability, accumulation of mutant organelles and cells, leading ultimately to malignant transformation [57].

Apart from inflammation, p62 has emerged as a crucial factor for HCC development. More specifically, ubiquitin-binding protein p62, also known as SQSTM1, is an intracellular adaptor protein involved in macroautophagy by playing a crucial role in the transfer of autophagic cargo to the autophagosome [58]. In addition, p62 has been recognized as a mediator for selective macroautophagy, as it can specifically recognize ubiquitinated proteins through a UBA domain and mediate interaction with LIR motif allowing bridging with LC3-II protein on the autophagosomes, contributing to translocation of the autophagic substrate to the lysosomes for degradation [59]. During lysosomal fusion, p62 is degraded together with the autophagic cargo; thereby, the cellular levels of this receptor are downregulated during macroautophagy activation [60].

The tumor-suppressive role of macroautophagy has been further proved by studies in HCC liver tissue, where macroautophagy defects have been widely observed and investigated as a crucial underlying pathogenic mechanism. Due to deficient macroautophagy, cancer hepatocytes exhibit increased oxidative stress and accumulation of p62, which is considered crucial for HCC development and malignant transformation [61]. Suppression of macroautophagy leads to accumulation of p62 and mutative proteins, otherwise degraded via lysosomes [62].

Intriguingly, p62, apart from being an autophagy flux biomarker, exerts multifaceted roles in cancer development. Chronic accumulation of p62 leads to constant activation of the nuclear factor kappa-light-chain-enhancer of activated B cells (NF-kB), the redox-sensitive transcriptional nuclear factor (erythroid-derived 2)-like 2 (NRF-2), and the mTOR pathway, thus preventing inflammation and oxidative stress while ensuring adequate energy levels for cancer cells, respectively. Accumulated p62 induces antioxidative response through NRF-2 transcription factor by competitively binding Keap1, thus preventing ubiquitination of NRF-2 [63,64]. Therefore, cancer hepatocytes tend to gain more oncogenic mutations without being subjected to oxidative stress response and cell-death inducing conditions. With the help of the unsuitable antioxidative ability, hepatocytes increase the risk of developing HCC [61]. In addition, p62 activates mTORC1, thereby promoting cell growth and proliferation, while concomitantly, p62 induces the expression of c-Myc, a widely known oncogenic protein, which promotes metabolic alterations and cellular proliferation [61]. Activation of NF-kB, a crucial inflammatory transcription factor, by p62 supports inflammation and cancer development [65], while concomitantly NF-kB acts as a promoter for further expression of p62 via a self-amplifying autoregulatory loop [66]. Apart from NF-kB, accumulated p62 also exacerbates inflammation through constant and unsuitable activation of other inflammatory molecules, such as NLRP3 inflammasome, which plays a vital role in the progression of preneoplastic lesions to HCC [61]. Thus, p62 plays a crucial and multifaceted role in the pathogenesis of HCC; thus, further studies are necessary to find out the complete underlying mechanism.

### 3.2. Upregulation of the CMA Pathway Promotes Tumor Cell Survival in Later Stages of HCC

CMA has a basal activity in all cells, along with macroautophagy, which cooperates to maintain cellular homeostasis. However, CMA activity is upregulated under certain stimuli, like substantial nutrient starvation, energy deprivation, or other severe stressful conditions [38]. Studies have shown increased expression of LAMP-2A, along with upregulation of co-localization of HSC-70 and LAMP-2A, thereby indicating increased CMA activity [67]. Compared to macroautophagy, upregulation of degradation levels of cytosolic proteins through the CMA pathway is mainly observed under prolonged periods of nutrient deprivation, as macroautophagy activity, which is the first to sense energy deprivation via the mTOR pathway, decreases subsequently [68]. On the contrary, CMA is progressively triggered in response to prolonged periods of energy deficits and long-term starvation conditions [38], which also characterize a tumor microenvironment.

Moreover, it has been demonstrated that CMA induction in liver cells under severe stress conditions is associated with degradation of Beclin 1, thereby suppressing autophagy at the level of initiation and autophagosome–lysosome fusion [69]. While a wide variety of research highlights the protective and tumor-suppressive role of macroautophagy, established information elucidates that HCC liver cells highly rely on autophagy pathways for survival. Recent evidence has reported that HCC development undergoes autophagy switching from a protective state characterized by high macroautophagy levels with low CMA levels to an HCC-promoting state characterized by low macroautophagy with high CMA levels [39]. During the later stages of carcinogenesis and since tumor cells are already established, autophagy flux is dysregulated and follows an unbalanced pattern, favoring tumor progression [70,71]. In fact, since elevated levels of p62 characterize HCC liver tissue, thus indicating macroautophagy impairment, new evidence has emerged highlighting that severe stressful stimuli, such as hostile tumor microenvironment, induce CMA activation [27].

In order to sustain their rapid proliferation, liver cancer cells confront increased energy demands; thus, CMA is activated as a response to prolonged stressful stimuli, thereby contributing to the survival of cancer cells in tumor microenvironments [72,73,74]. Various stressful stimuli have been associated with CMA activation, with prolonged starvation being the most common. In vivo and in vitro studies have shown that starvation activates CMA, although the exact mechanism has not been elucidated yet [38,75]. Oxidative stress also triggers CMA activity constitutively, as numerous studies have observed increased mRNA LAMP-2A levels and enhanced recruitment of LAMP-2A protein to the lysosomal membrane under oxidative stress [76,77,78,79].

Investigations in HCC nodules in the cirrhotic liver have shown increased expression of LAMP-2A, a marker of the CMA pathway. LAMP-2A is also considered an indicator of lysosome proliferation, used as a mechanism of increased cellular degradation and recycling of autophagic cargo to meet the elevated energy demands of cancer cells [28]. Moreover, a study in mice models by Ding et al. showed that CMA activation is required for HCC growth and is associated with recurrence of HCC [29]. The molecular and cellular mechanism of autophagy regulation and compensation between the two pathways is still under investigation. However, emerging evidence suggests that impaired macroautophagy and activation of CMA are associated with HCC development.

Moreover, highly stressful conditions associated with chronic HCV infection induce NRF-2 pathway activity, as an alternative way of cell survival, as it is considered a crucial antioxidant pathway. NRF-2 activation is mediated by the accumulation of p62, as mentioned above, and by increased oxidative stress, resulting in the induction of an antioxidative response, thereby promoting cell survival [80]. Moreover, NRF-2 has been shown to act as a transcription factor for key CMA molecules, such as LAMP-2A and HSC-70, enhancing their expression, thus acting as a potential mechanism of CMA activation under severe conditions stress conditions [69]. In addition, the NRF2 pathway modulates glucose metabolism through more efficient anabolic pathways for improving cell survival and tumor growth under stress [81].

Interestingly, mTORC2, the second complex of the mTOR signaling pathway, has been shown to inhibit CMA activation during HCV infection through AKT serine/threonine kinase 1 (AKT). Therefore, it should be further investigated how mTORC1 and mTORC2 complexes are inversely regulated and promote compensation between the two autophagic pathways under severe stress conditions and whether this cross-regulation underlies HCC development [82].

## 4. Autophagy and HCC Metastasis-Still a Matter of Discussion

Metastasis is an advanced stage of tumorigenesis and results in poor survival prognosis of the patients [83]. Metastasis is divided into several stages as it includes invasion of the tumor cell from the primary site, intravasation in the circulatory and lymphatic system, adherence of cancer cells to the vessel wall, extravasation of cancer cells at a distant site, and finally colonization of tumor cells at a destination organ [84]. Metastatic process triggers autophagy pathway, which plays a crucial role in the survival of metastatic cells in distant organs. However, as indicated in primary tumors, autophagy has a controversial role during the different stages of metastasis [85]. Notably, during the early stages of metastasis, autophagy suppresses cellular colonies trying to infiltrate distant organs.

On the other hand, in the advanced stages of metastasis, autophagy promotes the dissemination of cancer cells in the circulation [86] and contributes to the colonization of metastatic cells in the destination organ [87]. At the same time, it helps cells to survive in their new environment [88]. Interestingly, autophagy flux is upregulated when the metastatic site is established to promote cancer cell survival in new hostile conditions, such as hypoxia, nutrient deprivation, and detachment from the ECM [84,89]. Autophagy can also stimulate modifications in cell adhesion signaling that promote disassembly with adhesion molecules, thus contributing to the migration of malignant cells and invasion in distal organs [90].

Increased autophagic levels have been found in numerous metastatic cancers, including HCC, thus contributing to the aggregation of tumorigenesis [87]. However, whether the role of autophagy in HCC metastasis concerns macroautophagy or the CMA pathway remains to be elucidated; therefore, this review focuses only on the role of the general autophagy mechanism in HCC metastasis.

One study demonstrated that starvation-induced autophagy in HCC cells has been shown to facilitate metastasis by inducing mesenchymal expression, thereby promoting epithelial-mesenchymal transition (EMT), which is an essential intermediate stage of cancer metastasis [78,91]. Moreover, autophagy has been associated with cell invasion marker matrix metalloproteinase-9 (MMP-9) [92]. On the other hand, autophagy inhibition in HCC via silencing core macroautophagy proteins, such as Beclin 1 and ATG5, decreased distal metastasis to the lungs due to anoikis, a type of cell death induced in response to ECM detachment [87]. A variety of evidence has shown upregulated levels of LC3 in metastases, significantly higher than in patients with primary HCC [93].

## 5. Autophagy Mechanism Involved in HCC Therapy Resistance

Currently, HCC is the most common primary liver cancer, and according to the World Health Organization (WHO), HCC is the second leading cause of cancer-related death worldwide [94]. Prognosis remains poor as most of the patients with HCC have a 3 year survival rate of approximately 12.7%, and the median survival time is only 9 months [95]. To date, the most effective treatment for HCC is surgical resection, liver transplantation, or radiofrequency ablation for early and selected cases of intermediate stage HCC according to Barcelona Clinic Liver Cancer (BCLC) classification and treatment strategy criteria. Surgical management offers long-term survival with good quality of life [96,97]. However, the current therapeutic options regarding the advanced stage are multikinase inhibitors, such as sorafenib, regorafenib, or a combination of immunotherapy and anti-angiogenetic therapy, with expected survival ranging from 10 to 14 months [98]. Therefore, it is imperative to find new effective therapeutic approaches mainly for patients with advanced-stage HCC, whose current therapeutic options are restricted.

The autophagy mechanism seems to be a cardinal pathway underlying HCC development, as when the tumor is established, an increased autophagy flux is necessary to maintain cellular homeostasis and HCC development [99]. Therefore, managing the autophagic pathway may constitute a promising therapeutic approach, especially in patients who cannot undergo surgery.

Autophagy in the treatment of HCC is context dependent. Autophagy is considered a major mechanism that significantly affects the cellular response to anticancer drugs. More specifically, overstimulation of autophagy could exhibit cytotoxic properties via induction of self-digestion of cancer cells and therefore promoting of autophagic cell death, a well-established form of programmed cell death [100]. In fact, numerous chemotherapeutic drugs exert their anticancer effects by triggering autophagic cell death. Moreover, upregulation of the autophagy flux may implicate suppressing specific other cancer therapeutic targets to help against cancer, such as tumor invasion, migration, and tumor angiogenesis. On the contrary, autophagy could potentially act as a cytoprotective mechanism through the induction of drug resistance which constitutes a significant clinical obstacle to successful cancer treatment, leading to unsatisfactory survival rates of the patients [100]. Inhibition of treatment-induced autophagy has recently emerged as a potential promising therapeutic approach to enhance cancer therapy efficiency. Studies have shown that the combination of antineoplastic agents, along with the integration of autophagy inhibitors, could enhance the susceptibility of cancer cells to chemotherapeutic drugs that induce autophagy related resistance.

It is well demonstrated that autophagy could promote drug resistance by supporting the survival of tumor cells under metabolic stress induced by chemotherapy. Various chemotherapeutic drugs for HCC treatment could enhance autophagy flux in tumor cells, thereby contributing to drug resistance and promoting cell survival [101].

Chemotherapeutic drugs induce damage of various cellular components, such as accumulation of misfolded proteins, damaged DNA, and damaged organelles. However, instead of promoting cell death of cancer cells, these molecules could be degraded to substances that support metabolism and promote further tumor cell growth and survival through the autophagy pathway, thereby promoting resistance to therapeutic drugs. The resistance mechanism could be divided into two categories, the first one associated with high basal autophagy flux in some kinds of tumor cell types, resulting in intrinsic resistance to chemotherapy, and the second one associated with gradual increasing autophagic flux in response to long-term chemotherapy, thus promoting acquired drug resistance [101].

Both autophagy inducers and inhibitors have been proposed as effective treatment approaches. More specifically, sorafenib, the first-line drug used for the treatment of advanced stage HCC, may act as an autophagy inducer molecule. Sorafenib has been shown to promote cell death and inhibition of tumor growth by promoting cell cycle arrest. However, it has also been associated with the upregulation of autophagy via different mechanisms, such as the inhibition of PI3K/AKT/mTOR [102]. Thus, apart from its tumor-suppressing function, long-term treatment with sorafenib has been proven to trigger chemoresistance in HCC cells by induction of autophagy and promotion of tumor cell survival. In addition, sorafenib can inhibit tumor angiogenesis, thereby exacerbating HCC hypoxia. The hypoxic state of HCC is one of the primary triggers for constitutive induction of autophagic flux, thus acting as a mechanism of chemotherapeutic resistance [103]. At this point, autophagy inhibitors can play a key role in inhibiting chemoresistance since they can increase the sensitivity of cancer cells to therapeutic agents that induce autophagy. According to studies apart from autophagy inhibitors, the combination of sorafenib with other autophagy-inducers has been shown to be more effective against HCC development compared to treating with sorafenib alone [104]. In addition, the combination of melatonin with sorafenib has been demonstrated to enhance sorafenib’s cytotoxicity against human HCC cells by decreasing hypoxic resistance. Considering the constantly increasing occurrence of tumor resistance, the combination of different autophagy-related drugs with sorafenib are currently under investigation as a new therapeutic approach, which could enhance chemo-sensitivity and inhibit tumor cell survival.

According to research, chemoresistance could be abrogated by the inhibition of autophagy via genomic interference against autophagic genes, such as small-interfering RNA (siRNA) targeting core autophagic proteins, such as ATG3, ATG5, ATG7, and Beclin 1, or pharmacological inhibitors of critical components within the autophagy pathway [102]. In addition, it has been demonstrated that the Bcl-2 family of proteins are not only regulators of apoptosis signaling [103], but they could be used as effective autophagy inhibitors, thereby enhancing the effects of chemotherapeutic drugs and preventing the development of autophagy-related chemoresistance.

Moreover, core autophagic molecules may affect the sensitivity of HCC cells to chemotherapeutic drugs by interacting with apoptotic molecules, thus balancing between cell survival and cell death. Studies have shown that autophagic proteins may decrease proapoptotic molecules, such as Bad and Bim, thereby suppressing chemotherapeutic induced apoptosis, while on the other hand, suppression of autophagy may reverse HCC resistance to chemotherapy [76,105]. Furthermore, it has been demonstrated that Beclin 1, an autophagy related protein, may also regulate apoptosis through the interaction with multiple antiapoptotic Bcl-2 family proteins [105]. Activation of autophagy by chemotherapeutic drugs leads to the release of Beclin 1 from the Beclin 1-Bcl-2 complex, thereby increasing both autophagic flux and anti-apoptotic effects in tumor cells [101].

It is evident that further investigation is imperative to confirm that autophagy acts as an adaptive mechanism of resistance to anti-tumor drugs; therefore, inhibition of autophagy could further enhance the cytotoxic effects of these drugs synergistically. It is noteworthy that co-administration of microRNA (miR)-375, an inhibitor of autophagy with sorafenib, encapsulated together into calcium carbonate nanoparticles (miR-375/Sf-LCC NPs), has led to significant autophagy inhibition and enhanced anti-tumor effects of sorafenib in HCC [106]. Studies in preclinical models have well-established autophagy as a therapeutic target, with inhibition of autophagy being associated with enhanced chemo-sensitivity and promotion of tumor cell death. Targeting autophagic flux by combining autophagy inhibitors with cytotoxic chemotherapy is a promising therapeutic strategy for HCC. A recent report has demonstrated that CD133 monoclonal Ab could increase the susceptibility of HCC cells to doxorubicin and cisplatin by inhibiting autophagy and promoting cancer cell death [100]. Therefore, targeting CD133 with the autophagy inhibitor CD133 monoclonal Ab could be considered as a promising therapeutic approach for HCC.

Furthermore, a recent study showed that GNS561, a new autophagy inhibitor, exerted potent anti-tumor activity against a panel of tumor cancer cell lines and in two hepatocellular carcinomas in vivo models [107]. A multicenter, phase 1/2a study is currently running for patients with advanced primary and secondary liver cancer [107].

Currently, the available data on autophagy-related therapy combined with conventional chemotherapeutic drugs have emerged only from preclinical models, without further information about human liver tissue. Nonetheless, preclinical data suggest that targeting autophagy could be a promising therapeutic approach. Apart from conventional chemotherapy, autophagy could be combined with other management strategies, such as transarterial chemoembolization to target residual tumor cells [108].

## 6. Conclusions

HCC, accounting for 90% of primary liver malignancies, is a leading cause of cancer-related death, with an estimated incidence of >1 million cases by 2025 [109]. Unfortunately, patients are mainly diagnosed at an advanced stage, with a poor prognosis [95] and limited effective therapeutic approaches [110]. Intriguingly, the autophagy mechanism has been elucidated as a crucial regulator of HCC development with a complex and controversial role. However, recent studies have gained further insight into this mechanism, and they have shown that not only macroautophagy (usually referred to as “autophagy” in previous studies) but also the CMA pathway is deeply involved in HCC growth [29,69]. Macroautophagy seems to be the first sensor of stressful stimuli; therefore, it is probably upregulated during the early stages of carcinogenesis [39]. The role of macroautophagy in the degradation of aggregated proteins and dysfunctional organelles, along with anti-inflammatory properties and inhibition of accumulation of p62 protein, seems to act as a tumor suppressor mechanism leading to maintenance of hepatic homeostasis and genomic stability [78]. On the other hand, when the stressful stimuli are prolonged, the accumulation of dysfunctional cytosolic components over-exceeds macroautophagy capacity to eliminate harmful cellular components, leading eventually to impairment of the autophagic flux. Thus, CMA seems to be upregulated when the tumor is established as a potential compensatory mechanism to impaired macroautophagy [27]. Tumor cells, which are surrounded by a hostile microenvironment, could probably use the CMA pathway as a cell survival mechanism, thus promoting HCC development. CMA is responsible for the degradation of 30% of cytosolic proteins; thus, it is considered a significant mediator of amino-acids pool enrichment, especially under severely stressful conditions [38]. Up to date, there is not enough evidence on whether CMA and macroautophagy act synergistically or antagonistically and what conditions stimulate their relationship. However, a recent study has shown that CMA may inhibit macroautophagy through the degradation of Beclin 1, thereby resulting in p62 accumulation, a characteristic feature of liver cancer nodules. P62 subsequently acts as a crucial regulator of HCC development by promoting antioxidant pathways (NRF-2), cell proliferative mechanisms (mTOR), and inflammatory conditions (NF-kB), thus contributing to tumor cell growth [61].

Intriguingly numerous studies have explored factors that could potentially regulate levels of autophagic flux. Recently, long-noncoding RNAs (lncRNAs) have elucidated as potential regulators of autophagic levels that could lead to HCC development or HCC suppression. For instance, the lncRNA HULC, which is specifically overexpressed in HCC, could activate autophagy, thereby promoting HCC development, while concomitantly reducing chemosensitivity of cancer cells [111,112]. On the contrary, other lncRNAs, such as the phosphatase and tensin homolog pseudogene 1 (PTENP1), which has been found downregulated in HCC, could act as tumor suppressors by activating autophagy and promoting autophagic cell death of tumor cells and inhibiting migration, invasion, and angiogenesis [113]. These controversial results seem to correlate with the respective tumor-promoting and tumor-suppressing role of autophagy flux; however, lncRNAs are still at a very early stage and further studies are imperative to investigate their relationship with macroautophagy and CMA pathway. The discovery of the complete interaction mechanism would crucially contribute to the research of autophagy-related regulatory factors of hepatocarcinogenesis and be potentially considered as a promising therapeutic target. Interestingly, expression patterns of several lncRNAs have been proposed recently as a type of “signature biomarkers” that could have a prognostic value in overall survival and disease-free survival rates of patients with HCC [54,113,114].

Taken together, the crucial role of autophagy in HCC pathogenesis is more than evident; however, further studies are needed to investigate the roles of macroautophagy and CMA both in early and in the advanced stages of carcinogenesis and the molecular mechanisms that underlie their cross-regulation and the switching of the autophagy flux from a cancer-protective to a cancer-promoting role.

Regarding the role of autophagy in the management of HCC, there is accumulating evidence suggesting that the combination of antineoplastic agents and autophagy inhibitors increases cancer cells’ susceptibility and combat chemoresistance in a synergistic action. Therefore, autophagy modulation can be a promising way for more effective HCC management in the future. However, further investigation of autophagy related therapeutic approach is imperative, as inhibition of autophagy in dying-tumor cells could also inhibit their immune-mediated clearance, as autophagy is necessary for effective T-cell response [109].
